# Depression, Metabolic Syndrome, Serum TSH, and Vitamin D Concentrations in Rural and Urban Postmenopausal Women

**DOI:** 10.3390/medicina56100511

**Published:** 2020-09-30

**Authors:** Iwona Bojar, Dorota Raczkiewicz, Beata Sarecka-Hujar

**Affiliations:** 1 Department of Women’s Health, Institute of Rural Health, 20-950 Lublin, Poland; iwonabojar75@gmail.com; 2 Institute of Statistics and Demography, Collegium of Economic Analysis, SGH Warsaw School of Economics, 02-554 Warsaw, Poland; dorota.bartosinska@gmail.com; 3 Department of Basic Biomedical Science, Faculty of Pharmaceutical Sciences in Sosnowiec, Medical University of Silesia in Katowice, 41-200 Sosnowiec, Poland

**Keywords:** depression, TSH, metabolic syndrome, vitamin D, post menopause

## Abstract

*Background and objectives:* Depression is a serious problem affecting people worldwide, however it more commonly concerns women. Depression reduces the quality of life and, in many cases, leads to suicide. Numerous new biological factors have been demonstrated to have an impact on the pathogenesis of depression, including vitamin D, thyroid hormones, as well as factors related to heart disease. The aim of the study was to assess the impact of serum thyroid stimulating hormone (TSH) and vitamin D concentrations as well as metabolic syndrome on the severity of depression in Polish postmenopausal women from urban and rural areas. *Materials and Methods:* The study was conducted in 2018–2019 in the Lublin region, Poland, and comprised 396 postmenopausal women (239 living in rural areas and 157 living in urban areas). Metabolic syndrome criteria according to the International Diabetes Federation and Beck Depression Inventory were used, and laboratory blood tests were performed. *Results:* A significantly higher percentage of the examined rural residents had moderate or severe depression in comparison to the urban ones (*p* = 0.049). The examined women from rural areas had a significantly higher serum vitamin D concentration in comparison to the urban ones (*p* < 0.001). The rural residents more commonly had below-normal levels of serum TSH and less commonly had normal levels in comparison to the urban residents. Metabolic syndrome was found in 70% of the rural residents, and that number was significantly lower in the urban ones (22%, *p* < 0.001). *Conclusions:* The severity of depression in postmenopausal Polish women was correlated negatively with the serum TSH concentration in women from rural areas. The severity of depression was increased in urban postmenopausal women with hypertension. No correlation of the depression severity with the serum vitamin D concentration or other criteria of metabolic syndrome was found.

## 1. Introduction

As the most prevalent psychiatric disorder, depression is a serious problem affecting people worldwide. Despite the existence of effective treatment, in many countries about 80% of patients with mood disorders remain untreated. Depression is more commonly observed in women; it reduces the quality of life and, in many cases, leads to suicide [1]. In an Australian study, women in perimenopausal and postmenopausal stages were found to have a higher risk of more severe symptoms of depression than women in the premenopausal stage without a history of depression [2].

Numerous aspects of neural function influence the pathogenesis of depression. Previously, several new biological factors have been demonstrated to correlate with depression, especially in combination with more traditional mechanisms. One of these new factors is the level of vitamin D, which was linked to both unipolar and bipolar depression [3]. However, until now this association has been unclear and the available data are contradictory [4,5,6]. In 2005, Eyles et al. [7] reported that the human brain contains receptors for vitamin D as well as an 1a-hydroxylase (1a-OHase), both of which were found in neurons and glial cells. Thus, the authors suggested that vitamin D may have autocrine/paracrine properties in the human brain [7]. Cui et al. [8], in an in vitro study on human SH-SY5Y cells, demonstrated that vitamin D increased tyrosine hydroxylase expression, which is the rate-limiting enzyme in dopamine synthesis. Moreover, according to the monoamine hypothesis, the pathophysiological basis of depression is the reduction in the levels of serotonin, norepinephrine, and/or dopamine in the central nervous system [9]. In addition, Cui et al. [8] found also that vitamin D elevated N-cadherin, which is a mediator of the regulation of tyrosine hydroxylase expression.

Depressive symptoms were observed to be high in hypothyroidism, since the thyroid hormones play a crucial role in brain development [10]. Some of the researchers use the theory of “brain hypothyroidism”, in which depression is caused by local hypothyroidism in the brain, with a simultaneous normal level of thyroid hormone peripherally. Patients with depression have alterations in the function of the hypothalamic-pituitary-thyroid axis, including the loss of the nocturnal thyroid stimulating hormone (TSH) rise [11]. In addition, several clinical features of hypothyroidism can be found in depression.

Various studies have confirmed the correlation between depression and obesity [12,13,14]. Serum levels of TSH as well as the data provided by the Center for Epidemiological Studies-Depression (CES-D) scale were found to be positively correlated with BMI in a large group of centrally obese patients [12]. Additionally, a study by Mannan et al. [14] demonstrated a bi-directional correlation between obesity and depression. Adolescents diagnosed with depression had a 70% higher risk of being obese, whereas obese adolescents had a 40% increased risk of being depressed. This correlation was found to be stronger among female adolescents [14]. Both obesity and metabolic syndrome (MetS) predicted a poor outcome of depression in 273 major depressive Spanish patients (51 years old on average) over a period of 12 months [15], which may be a very important observation from a clinical point of view. A systematic review of studies regarding depression and MetS showed abdominal obesity, which is a component of MetS, to be associated more strongly and consistently with depression in people over 60 [16].

The aim of the present study was to assess the impact of the serum TSH and vitamin D concentrations as well as metabolic syndrome, on the severity of depression in Polish postmenopausal women from urban and rural areas.

## 2. Materials and Methods

### 2.1. Study Groups

The study was conducted in 2018–2019 in the Lublin region, Poland, and comprised 396 postmenopausal women (239 living in rural areas and 157 living in urban areas). The inclusion criteria were age 45–65, and at least 12 months from the last menstruation. The exclusion criteria were active cancerous disease within 5 years after recruitment; a medical history of mental diseases; addiction to drugs and/or alcohol; diagnosed disease entity with symptoms of dementia; current or past use of HRT; and severe menopausal symptoms according to the Kupperman scale. At the initial stage of the study, when the volunteers were being qualified, a brief Montreal Cognitive Assessment test was performed in order to include in the study women who did not show the features of dementia. Women who obtained scores of at least 26 were included in the study.

Participation in the survey was voluntary, and informed consent for participation was obtained from all the volunteers.

The study was approved by the Ethics Committee of Institute of Rural Medicine in Lublin, Poland.

### 2.2. Laboratory Blood Tests

Antecubital venous blood (10 mL) was collected after overnight fasting from all the recruited women. The samples were centrifuged within 2 h after being drawn to obtain serum. Laboratory tests were performed in an accredited Laboratory of Medical Analyses ALAB (Lublin, Poland) to measure the following parameters: the fasting glucose concentration and lipid profile components—i.e., total cholesterol (Total CHOL), high-density lipoprotein cholesterol (HDL-CHOL), triglycerides (TG), serum TSH, and vitamin D concentrations. The low-density lipoprotein cholesterol (LDL-CHOL, mg/dl) was calculated based on the Friedewald formula: LDL = total cholesterol—(HDL cholesterol)—(TG/5). The thyroid stimulating hormone (TSH)** level was measured by the chemiluminescence immunoassay (CLIA) method using the ATELLICA IM1600 analyzer. The vitamin D level was measured by the CLIA method using the LIAISON XL analyzer. The lipid and glucose concentrations were measured by enzymatic methods using an ADVIA Chemistry System analyzer.

The standard limits of the serum TSH concentration were: below normal, <0.55 mlU/L; normal, 0.55–4.78 mlU/L; above normal, >4.78 mlU/L.

The standard limits of serum vitamin D concentration were: severe deficiency, <10 ng/mL; moderate deficiency, 10–20 ng/mL; mild deficiency, 20–30 ng/mL; optimal, 30–80 ng/mL.

### 2.3. Determining the Metabolic Syndrome

The presence of MetS according to the International Diabetes Federation was determined in the examined women. MetS was defined as the coexistence of at least 3 of the following 5 risk factors:

(1) Waist circumference ≥80 cm;
(2) TG ≥150 mg/dL or treatment for dyslipidemia;
(3) HDL <40 mg/dL or treatment for dyslipidemia;
(4) Systolic blood pressure ≥130 mm Hg and/or diastolic blood pressure ≥85 mm Hg or antihypertensive therapy;
(5) Fasting glucose ≥100 mg/dL or hypoglycemic treatment.

Blood pressure was measured in the morning sitting down after 15 min rest with a standardized blood pressure monitor.

### 2.4. Determining the Severity of Depression

The Beck Depression Inventory (BDI) by Aaron Beck consists of 21 items. Respondents provide answers independently, choosing statements which best describe their well-being. The level of depression is determined by summing up the number of scores obtained: lack of depression or minimal (0–10 score), moderate depression (11–27 score), and severe depression (28 score or more).

### 2.5. Statistical Methods

The data were statistically analyzed using the STATISTICA 13 software. The mean and standard deviation were estimated for continuous variables, as well as absolute numbers (*n*) and percentages (%) of the occurrence of items for categorical variables.

The following statistical tests were used:

- Pearson’s chi-square test to compare the categorical variables between the rural and the urban residents.
- Student’s *t*-test to compare the continuous variables between the rural and urban residents, to compare the severity of depression between the women with and without MetS, to compare the severity of depression between the women with the fulfilled criterion of metabolic syndrome and those not fulfilled.
- Pearson’s correlation coefficient to correlate the severity of depression with the serum vitamin D and TSH concentrations, as well as with the number of MetS fulfilled criteria.
- The significance level was assumed to be 0.05.

## 3. Results

### 3.1. Characteristics of Study Groups

The examined women living in rural areas were 56.8 years old on average, while those living in urban areas were 56.4 years on average (Table 1). The urban residents were significantly older at the last menstruation time than the rural residents (50.3 vs. 49.0 years on average, *p* = 0.003). The rural residents were more obese than the urban ones (BMI 29 vs. 26, *p* < 0.001). Obesity was found in 18% of the urban residents, whereas it was significantly more common in the rural residents (36%, *p* < 0.001). The women from urban areas were significantly better educated than the rural ones (*p* < 0.001). The urban residents more commonly had never been married or were divorced and were less commonly widowed than the rural ones. A total of 95% of the examined rural residents and 92% of the urban ones had children.

### 3.2. Severity of Depression in Study Groups

**Figure 1:**
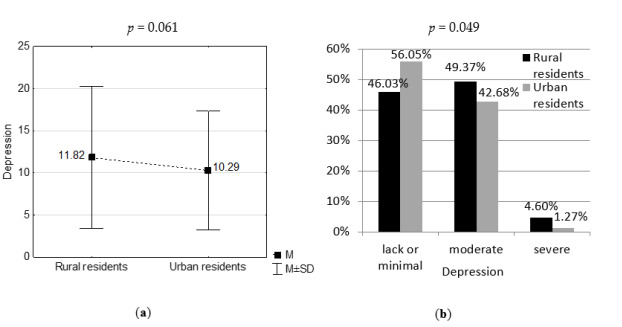
Severity of depression in the study groups: (**a**) in scores, (**b**) in intervals. M—mean, SD—standard deviation.

The severity of depression according to the Beck Depression Inventory was 12 on average in the rural residents, while it was 10 on average in the urban residents (Figure 1). A significantly higher percentage of the examined rural residents had moderate or severe depression in comparison to the urban ones (*p* = 0.049).

### 3.3. Serum TSH, Vitamin D Concentrations, and Metabolic Syndrome in Study Groups

The examined women from rural areas had significantly higher serum vitamin D concentrations in comparison to the urban ones (*p* < 0.001). A moderate or severe deficiency of serum vitamin D concentration was more common in the urban residents compared to the rural ones (Table 2).

The rural residents more commonly had below-normal levels of serum TSH and less commonly had normal levels in comparison to the urban residents.

MetS was found in 70% of the rural residents, and that number was significantly lower in the urban ones (22%, *p* < 0.001). It was more common for the rural residents to suffer from abdominal obesity, hypertriglyceridaemia, arterial hypertension, and hyperglycaemia than the urban ones, whereas the prevalence of low HDL-cholesterol was comparable in both groups.

### 3.4. Correlations between Serum TSH, Vitamin D Concentrations, Metabolic Syndrome, and Severity of Depression in Study Groups

**Figure 2:**
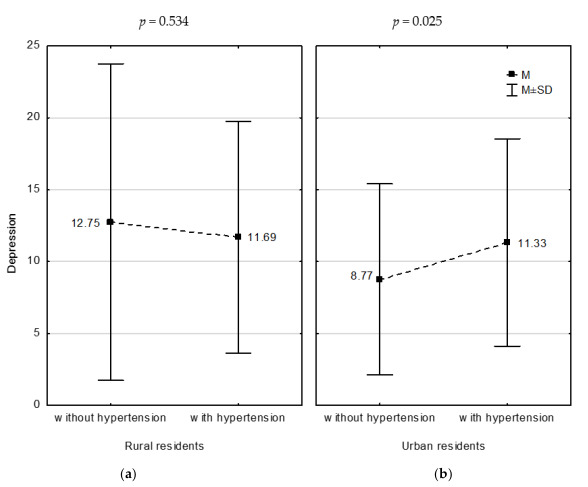
Severity of depression versus arterial hypertension in the study groups: (**a**) in rural residents, (**b**) in urban residents. M—mean, SD—standard deviation.

The severity of depression correlated negatively with the serum TSH concentration in the rural residents (r = −0.211, *p* = 0.049) and was significantly higher in the urban residents with hypertension compared to those without hypertension (Figure 2, *p* = 0.025). However, the severity of depression did not correlate with the serum vitamin D concentration, the prevalence of metabolic syndrome, the number of fulfilled criteria, and other criteria of metabolic syndrome (Table 3).

## 4. Discussion

According to the meta-analysis by Ferrari et al. [17], major depressive disorder is more prevalent among women than among men (5.8% vs. 3.5%, respectively). Due to the long life expectancy of an average woman, the postmenopausal period covers about one third of a women’s life. During this period, the risk of developing many diseases increases, which poses a considerable challenge in providing appropriate care and treatment for emerging ailments. The problem of depression in post-menopausal women affects all aspects of their daily functioning. An increased risk of depression during the menopause transition has been previously observed [18,19,20]. In a Chinese study based on a group of 430 women from urban areas, almost 20% of postmenopausal women had symptoms of depression, compared to 14.5% of premenopausal women [21]. Additionally, the independent predictors for symptoms of depression were as follows: higher body mass index, poor health, low education status, and night sweats [21]. A study based on Arab women also reported a greater prevalence of depression and anxiety in postmenopausal women compared to menopausal ones [22].

In our study, the frequency of moderate or severe depression was significantly higher in the women from rural areas than in the urban group. Similarly, in the study by Malacara et al. [23], a depressive mood in pre- and postmenopausal women was highly associated with rural residence. Rural post-menopausal women from India were also feeling depressed significantly more commonly than urban women [24].

As women inhabiting rural areas spend extended time outdoors, they are more exposed to the sun than women from urban areas. Thus, as expected, a moderate or severe deficiency of vitamin D levels was more prevalent in the urban female residents of our analyzed group. Similarly, the serum vitamin D concentration was significantly higher in the women from rural regions than in those from the urban areas. Our data are consistent with the results of a Malaysian study [25]. However, in our study the severity of depression did not correlate with the serum vitamin D concentration. The previous data concerning the impact of the level of vitamin D on depression have been inconclusive. Some authors have found a relation between the vitamin D level and depression, however others have not [5,26,27]. A meta-analysis on a sizeable group of over 31,000 patients indicated that cases with depression had lower vitamin D levels compared with the controls. The authors also observed that cohort studies indicated a significantly increased hazard ratio (HR = 2.21) of depression when a comparison of the lowest vs. highest vitamin D categories was performed [5]. On the other hand, a mendelian randomization study by Libuda et al. [26] did not reveal a significant correlation between vitamin D concentrations and depressive symptoms or bipolar depression. In turn, the median vitamin D levels in Turkish patients with major depressive disorder did not differ significantly from the control group (10.3 vs. 11.4 ng/mL) [27]. In addition, the authors did not find statistical differences in the genotype distribution for the rs2228570 (FokI) polymorphism of vitamin D between patients and controls [27].

A meta-analysis of nine studies comprising 4923 adults reported no significant reduction in depression after vitamin D supplementation [28]. Similarly, Marsh et al. [29] found that, in adults with bipolar depression and insufficient vitamin D levels, a significant increase in the concentration of vitamin D had no impact on the reduction in depression symptoms compared to the placebo group. The same was observed for women specifically. In the study by Mason et al. [30], when a placebo group and overweight or obese postmenopausal women were given a 2000 IU vitamin D supplementation daily for 12 months, no significant change in depressive symptoms was observed.

Our study revealed that the severity of depression was significantly higher in the urban residents with hypertension compared to those without hypertension. In addition, we observed no correlation between the severity of depression and the serum vitamin D concentration, as well as the criteria of MetS. In the analyzed group of women, a significant difference in the prevalence of MetS among rural and urban was demonstrated (70% vs. 22%, respectively). Abdominal obesity, hypertriglyceridaemia, arterial hypertension, as well as hyperglycaemia characterized the rural women more commonly than the urban women. In the previous studies, vitamin D deficiency was observed in 60% of postmenopausal women with metabolic syndrome from Lithuania [31]. In a Korean study, vitamin D insufficiency was also found to be significantly associated with the presence of metabolic syndrome and hypertension in patients with psychotic disorders [32]. The previous data showed a link between MetS and major depressive disorder [33]. However, it is rather postulated that both disorders may cause one another. Patients with coronary heart disease (CHD) show a higher prevalence of depression [34], whereas, on the other hand, patients with depressive disorders are at an increased risk of MetS or CHD [35]. In middle-aged women who were free of the baseline MetS, current or previous major depression episodes were significantly associated with the onset and presence of MetS at the follow-up (odds ratio = 1.82) [35]. De Hert et al. [33] demonstrated an increased risk of developing coronary heart disease in cases with severe mental illness, including bipolar disorder or major depressive disorder, than in the controls (HR = 1.54). The results of a meta-analysis comprising 5531 patients showed a 1.5-fold higher prevalence of MetS in major depressive disorder compared to in the general population [36]. Previously, it was also suggested that the association of MetS and depression increased (two- to three-fold) the risk of cardio-vascular mortality in patients with major depressive disorder [37].

In our study, it was more common for the women from rural areas to have below-normal levels of serum TSH than for the urban women. In contrary, a normal level of TSH was less common in the rural women when compared to the urban ones. We also found that the severity of depression correlated negatively with the serum TSH concentration in rural women. It is postulated that depression should be considered in patients with a dysfunction of their thyroid, while depressed patients should be tested for TSH [38]. Data regarding the association between TSH and depression in postmenopausal women are scarce. In addition, this correlation is not fully understood, however the possible role in disease etiology was assigned to the changes in the catecholamine system of the hypothalamus–pituitary–thyroid axis, as well as in the serotonergic pathway [11,39]. A study by Guimarães et al. [40] based on almost 1300 Brazilian, middle-aged women from urban areas demonstrated that women with a high TSH level (>10 mUI/mL) had a three-fold higher chance of presenting depressive symptoms than women having with levels of TSH. In contrary, Loh et al. [41] reported no significant difference in the mean TSH level between individuals with depression and healthy controls (2.30 vs. 2.13 mIU/L, respectively).

Many confounding factors (e.g., access to healthcare or diet) may cause differences in the severity of depression and the prevalence of metabolic syndrome between rural and urban postmenopausal women. A study by Prättälä R. et al. [42] demonstrated some differences in diet between rural and urban residents. Rural inhabitants in Estonia and Finland ate meat more often than the urban ones. On the other hand, in Latvia and Lithuania no difference was found in meat consumption according to the place of residence. In turn, urban residents from Baltic countries more frequently consumed fruit on a daily basis, whereas in Finland fruit consumption did not vary by place of residence [42]. Rural women have been demonstrated to consume a significantly higher amount of protein, fat, saturated fat, or cholesterol in comparison to urban women of reproductive age from Australia [43]. Similarly, in postmenopausal women from Iran, women from rural regions showed a significantly higher intake of, among other things, calories, protein, total fat, or saturated fat than urban women [44].

In Poland, access to a primary healthcare is relatively wide, both for rural and urban residents. However, urban residents have much easier access to specialists, in contrast to rural ones. Primary healthcare services carries out initial diagnoses of the level of blood glucose, the serum lipid profile, and blood pressure. In order to improve the health situation of rural residents, more attention should be paid to health education, especially for rural women. It is vital for them to know that they can make an appointment with their primary healthcare physician, even without disease symptoms, just for preventive examinations. In Poland, employees of plants or large enterprises (most often located in urban areas) have access to preventive examinations. However, there are no health programs for rural residents, and thus it would be important to consider introducing mandatory preventive examinations for them. Health promotion is also of great importance, as our study shows that rural women obtained worse results for metabolic syndrome and severity of depression than the urban women did.

Our research may be important for public health and health promotion in Poland. In our opinion, the present results indicate that attention should be paid to preventive health care, including promoting proper dietary habits and preventive examinations for women from rural regions. Primary healthcare physicians working in rural health centers should also be involved in health promotion and health education. Decision-makers in Poland should consider introducing mandatory prophylactic programs for rural residents, the vast majority of whom are farmers, which exist for hired workers.

The present study has some limitations. Despite the fact that the women from both the rural and urban areas were age-matched, the number of urban women was lower. There are many other factors that may potentially affect the severity of depression in postmenopausal women. Further research with an increased sample size and with more confounding factors is required. We are aware of the impact of proper diet and other healthy lifestyle choices on the well-being and health outcomes of postmenopausal women. Thus, we intend to perform a comparison study on healthy habits, including proper nutritional diet, preventive behaviors, positive psychological attitudes, and health practices, between rural and urban postmenopausal women in the near future.

## 5. Conclusions

The severity of depression in postmenopausal Polish women was correlated negatively with the serum TSH concentration in the women from rural areas. The severity of depression was increased in urban postmenopausal women with hypertension. However, the depression severity did not correlate with the serum vitamin D concentration and the criteria of MetS apart from hypertension.

## Figures and Tables

**Table 1:** Study groups’ characteristics.

Parameter	Rural Residents (*n* = 239)	Urban Residents (*n* = 157)	Comparison between Rural and Urban	
			Test ^1^	*p*
Age (years), M ± SD	56.82 ± 4.40	56.38 ± 3.34	1.072	0.285
Age at last menstruation (years), M ± SD	48.95 ± 4.22	50.27 ± 4.08	−3.016	**0.003**
BMI (kg/m^2^), M ± SD	28.83 ± 5.01	26.36 ± 4.36	5.045	**<0.001**
normal weight	56 (23.43)	65 (41.40)	21.271	**<0.001**
overweight	96 (40.17)	64 (40.76)		
obesity	87 (36.40)	28 (17.83)		
Level of education, *n* (%)				
primary	27 (11.29)	1 (0.64)	168.621	**<0.001**
basic vocational	104 (43.51)	5 (3.18)		
secondary	97 (40.59)	65 (41.40)		
tertiary	11 (4.60)	86 (54.78)		
Marital status, *n* (%)				
married	204 (85.36)	128 (81.53)	24.504	**<0.001**
never married	3 (1.26)	7 (4.46)		
divorced	4 (1.67)	16 (10.19)		
widowed	28 (11.72)	6 (3.82)		
Having children, *n* (%)	228 (95.40)	144 (91.72)	2.251	0.134

^1^ Student’s *t*-test for continuous variables or chi-square test for categorical variables. Significant differences are in bold. M—mean, SD—standard deviation, BMI—body mass index.

**Table 2:** Serum TSH, vitamin D concentrations, and metabolic syndrome in the study groups.

Parameter	Rural Residents (*n* = 239)	Urban Residents (*n* = 157)	Comparison between Rural and Urban	
			Test ^1^	*p*
Vitamin D (ng/mL), M ± SD	22.79 ± 8.01	17.31 ± 8.79	6.402	**<0.001**
severe deficiency, *n* (%)	7 (2.93)	32 (20.38)	48.650	**<0.001**
moderate deficiency, *n* (%)	90 (37.66)	77 (49.04)		
mild deficiency, *n* (%)	95 (39.75)	32 (20.38)		
optimal, *n* (%)	47 (19.67)	16 (10.19)		
TSH (mIU/L), M ± SD	1.46 ± 1.28	1.55 ± 0.98	−0.759	0.448
below normal, *n* (%)	40 (16.74)	13 (8.28)	6.832	**0.033**
normal, *n* (%)	193 (80.75)	142 (90.45)		
above normal, *n* (%)	6 (2.51)	2 (1.27)		
Metabolic syndrome, *n* (%)	168 (70.29)	34 (21.66)	89.695	**<0.001**
Number of fulfilled criteria of metabolic syndrome, *n* (%)				
0	2 (0.84)	18 (11.46)	106.489	**<0.001**
1	15 (6.28)	47 (29.94)		
2	54 (22.59)	58 (36.94)		
3	110 (46.03)	25 (15.92)		
4	41 (17.15)	7 (4.46)		
5	17 (7.11)	2 (1.27)		

^1^ Student’s *t*-test for continuous variables or chi-square test for categorical variables. Significant correlations are in bold. M—mean, SD—standard deviation, TSH—thyroid stimulating hormone.

**Table 3:** Correlations of the depression severity with the serum TSH, vitamin D concentration, and metabolic syndrome in study groups.

Parameter	Test ^1^	Rural Residents (*n* = 239)		Urban Residents (*n* = 157)	
		Result	*p*	Result	*p*
Vitamin D (ng/mL)	r	0.062	0.343	0.010	0.900
TSH (mIU/L)	r	−0.211	**0.049**	0.060	0.487
Metabolic syndrome	t	−0.083	0.934	0.759	0.449
Number of fulfilled criteria of metabolic syndrome	r	0.020	0.765	0.051	0.524
abdominal obesity	t	0.258	0.796	0.788	0.432
hypertriglyceridaemia	t	−0.423	0.671	0.273	0.785
low HDL-cholesterol	t	−0.459	0.647	1.350	0.179
arterial hypertension	t	0.623	0.534	−2.264	**0.025**
hyperglycaemia	t	−0.224	0.823	0.729	0.467

^1^ r—Pearson’s correlation coefficient; *t*—Student’s *t*-test. Significant correlations are in bold. TSH—thyroid stimulating hormone, HDL—high-density lipoprotein cholesterol.
